# Genomic Analysis of *Klebsiella pneumoniae* ST258 Strain Coproducing KPC-2 and CTX-M-14 Isolated from Poultry in the Brazilian Amazon Region

**DOI:** 10.3390/antibiotics11121835

**Published:** 2022-12-17

**Authors:** Tiago Barcelos Valiatti, Rodrigo Cayô, Fernanda Fernandes Santos, Francisco Ozório Bessa-Neto, Ramon Giovani Brandão Silva, Ruanita Veiga, Márcia de Nazaré Miranda Bahia, Lívia Maria Guimarães Dutra Guerra, Antônio Carlos Campos Pignatari, Cintya de Oliveira Souza, Danielle Murici Brasiliense, Ana Cristina Gales

**Affiliations:** 1 Laboratório Alerta, Disciplina Infectologia, Departamento de Medicina, Escola Paulista de Medicina (EPM), Universidade Federal de São Paulo (UNIFESP), São Paulo 04039-032, SP, Brazil; 2 Laboratório de Imunologia e Microbiologia (LIB), Setor de Biologia Molecular, Microbiologia e Imunologia, Departamento de Ciências Biológicas (DCB), Instituto de Ciências Ambientais, Químicas e Farmacêuticas (ICAQF), Universidade Federal de São Paulo (UNIFESP), Diadema 09972-270, SP, Brazil; 3 Seção de Bacteriologia e Micologia, Instituto Evandro Chagas (IEC), Ananindeua 67030-000, PA, Brazil; 4 Laboratório Especial de Microbiologia Clínica (LEMC), Disciplina Infectologia, Departamento de Medicina, Escola Paulista de Medicina (EPM), Universidade Federal de São Paulo (UNIFESP), São Paulo 04025-010, SP, Brazil

**Keywords:** resistome, carbapenemase, genomic surveillance, one health, food-production animal, Brazilian Amazon Region

## Abstract

This study aimed to characterize a *Klebsiella pneumoniae* strain (KP411) recovered from the stool samples of poultry (*Gallus gallus*) in the Brazilian Amazon Region. The whole-genome sequencing of KP411 revealed the presence of an important arsenal of antimicrobial resistance genes to β-lactams (*bla*_CTX-M-14_, *bla*_TEM-1B_, *bla*_KPC-2_, *bla*_SVH-11_), aminoglycosides [*aph(3″)- Ib, aph(6)-Id, aph(3′)-Ia*], sulfonamides (*sul1, sul2*), quinolones (*oqxAB*), fosfomycin (*fosA*^KP^), and macrolides [*mph(A)*]. Furthermore, our analyses revealed that the KP411 strain belongs to the ST258 clonal lineage, which is one of the main epidemic clones responsible for the dissemination of KPC-2 worldwide. Our data suggest that food-producing animals may act as reservoirs of multidrug-resistant *K. pneumoniae* belonging to the ST258 clone, and, consequently, contribute to their dissemination to humans and the environment.

## 1. Introduction

Antimicrobial resistance (AMR) rates among the main pathogens of clinical importance have increased in the last decade worldwide, making AMR one of the main public health concerns. One of the factors that justify this phenomenon is the spread and establishment of international epidemic clones whose main characteristic is a large arsenal of antimicrobial resistance genes (ARGs), especially those encoding for carbapenemases [1,2]. In this context, the indiscriminate prescription of broad-spectrum cephalosporins, especially in the 1980s and 1990s, contributed to the emergence of extended-spectrum β-lactamases (EβLs), thus limiting the use of this antimicrobial in clinical practice and leading to the increase of carbapenem usage. Shortly thereafter, carbapenemases (enzymes capable of hydrolyzing carbapenems) have emerged as one of the most important mechanisms of resistance to β-lactams, impairing the use of carbapenems, considered one of the last resorts for the treatment of serious infections caused by Gram-negative bacilli (GNB) [3,4,5].

To date, *Klebsiella pneumoniae* carbapenemase (KPC) is the most “successful” carbapenemase described, which has been disseminated in healthcare settings worldwide, mainly among *K. pneumoniae* isolates [6,7]. As AMR rates are higher in the nosocomial environment, many studies sought to understand the spread of this multidrug-resistant (MDR) bacteria from hospitals to the environment. Previous studies revealed that AMR was present in all ecological niches, showing that the chain of dissemination of this phenomenon is interconnected and that it is increasingly necessary to adopt surveillance studies that contemplate the One Health perspective [8,9]. In Brazil, there is still an important gap regarding the monitoring of AMR in farm animals, especially in less populated geographic regions of the country, such as the Brazilian Amazon. Considering that food-producing animals play an important role as reservoirs of MDR isolates [8,9], this study aimed to characterize a *K. pneumoniae* strain to the sequence-type (ST)-258-carrying *bla*_KPC-2_ and other resistance genes recovered from poultry (*Gallus gallus*) in the Brazilian Amazon Region. 

## 2. Results and Discussion

The KP411 strain exhibited high-level resistance to all B-lactams, fluoroquinolones, and gentamicin (Table 1). In contrast, it was susceptible to amikacin (MIC, 4 μg/mL), colistin (MIC, 0.5 μg/mL), and polymyxin B (MIC, <0.25 μg/mL) (Table 1). Additionally, the KP411 strain showed a hypermucoviscous phenotype by the string test, which is often associated with hypervirulent *K. pneumoniae* strains. However, in the KP411 genome, it was not possible to identify the presence of the *rmpA* and *magA* (K1) genes, which are precursors of this phenotype [10,11]. Previous studies corroborate our findings, as they also observed the occurrence of hypermucoviscous phenotype in *K. pneumoniae* strains that did not carry such genes, reinforcing the hypothesis that there are other mechanisms involved in this virulence phenotype that has been described so far [12,13].

The genome assembly metric of the KP411 strain and the number of coding sequences (CDSs) annotated are shown in Table 2 The KP411 genome had a total size of 5,831,458 bp with 56.89% of G+C content and was distributed in 120 contigs. The N_50_ and N_75_ values were 199,121 and 128,838 bp, while L_50_ and L_75_ were 9 and 19, respectively. In addition, 15 rRNAs, 86 tRNAs, and 5881 coding sequences (CDSs) in the KP411 genome were found. Among the ARGs content, it was verified the presence of those that conferred resistance to β-lactams (*bla*_KPC-2_, *bla*_CTX-M-14_, *bla*_TEM-1B_, *bla*_SVH-11_), aminoglycosides [*aph(3″)-Ib*, *aph(6)-Id*, *aph(3′)-Ia*], sulfonamides (*sul1*, *sul2*), fosfomycin (*fosA^KP^*), quinolone (*oqxA*, *oqxB*), and macrolides (*mph(A)*, *erm(42))*. Point mutations in the *Gyr*A (S83I) and *Par*C (S80I) genes were also found and are known to be associated with fluoroquinolone resistance.

The *bla*_KPC-2_ gene is the main responsible gene for conferring resistance to carbapenems among *K. pneumoniae* clinical isolates in Brazil [14,15]. However, its occurrence in strains colonizing food-producing animals has not been fully unveiled in Brazil. According to the sequencing analyses, it was also verified that the KP411 strain belongs to ST258, KL107 (wzi:154), which is distinct from those considered more virulent for *K. pneumoniae* (K1 and K2), O2V2 lipopolysaccharide locus, and presented six different plasmid replicons: ColRNAI, IncC, IncFIB (K), IncFIB (pKPHS1), IncFII (K), and IncX3. Additionally, the analysis performed with the mlplasmids tool revealed that the contigs carrying *bla*_CTX-M-14_, *bla*_TEM-1B_, *bla*_KPC-2_, *aph(3″)-Ib*, *aph(6)-Id*, *aph(3′)-Ia*, *sul1*, *sul2*, and *mph(A)* genes were probably derived from plasmids. In addition, using this same tool, we found that IncX3 plasmid carried the *bla*_KPC-2_ gene in a genetic environment composed of IS*Kpn6*-*bla*_KPC-2_-*tnpA*-*tnpR*, which was curiously not associated with an entire Tn*4401* transposon, indicating the presence of a non-Tn*4401* element (NTE_KPC_) variant, as previously described [16]. The ST258 is largely related to the spread of KPC-2 worldwide, including Brazil [2,17,18], and has been classified as a high-risk clone, being frequently associated with severe infections in many countries with high-mortality rates [19]. As verified in our study, Yang et al. [20] also reported the occurrence of ST258 in poultry and swine in China, reinforcing that these animals can act as a reservoir of clinically important MDR clones.

Interestingly, the IncX3 plasmid found in the KP411 strain has been also associated with the spread of *bla*_KPC-2_ in *K. pneumoniae* ST258 strains in the United States [21] and Australia [22]. The alignment of the contig sequences containing the IncX3 replicon and the *bla*_KPC-2_ gene with other plasmids deposited in the GenBank revealed a high nucleotide identity (varying from 99.85% to 100%) with IncX3 plasmids isolated from *K. pneumoniae* and *Escherichia coli* strains recovered from different Brazilian states (Table 2), suggesting possible horizontal dissemination of conjugative plasmids harboring *bla*_KPC-2_. As far as we know, this is the first time that *K. pneumoniae* ST258-producing KPC and CTX-M-14 has been found in food-production animals in Latin America. To better understand this scenario, more antimicrobial-resistance surveillance studies should be carried out in this region to verify whether animals are acting as a reservoir for these MDR isolates or whether humans are transmitting these bacteria to animals. In addition, we believe that the environment plays an important role in this transmission route, especially in this region of the country, where most homes do not have access to sewage treatment.

Furthermore, to the best of our knowledge, this is also the first report of the *K. pneumoniae* strain carrying the plasmid IncX3-*bla*_KPC-2_ isolated from poultry, as, until now, all reported isolates were recovered from humans. In addition, a previous study indicates that IncX3 plasmids have acquired clinical importance worldwide, as they have already been associated with resistance mechanisms against different antimicrobial classes [23]. Interestingly, the KP411 strain also harbored the *bla*_CTX-M-14_ gene that was previously associated with MDR strains causing outbreaks.

**Figure 1:**
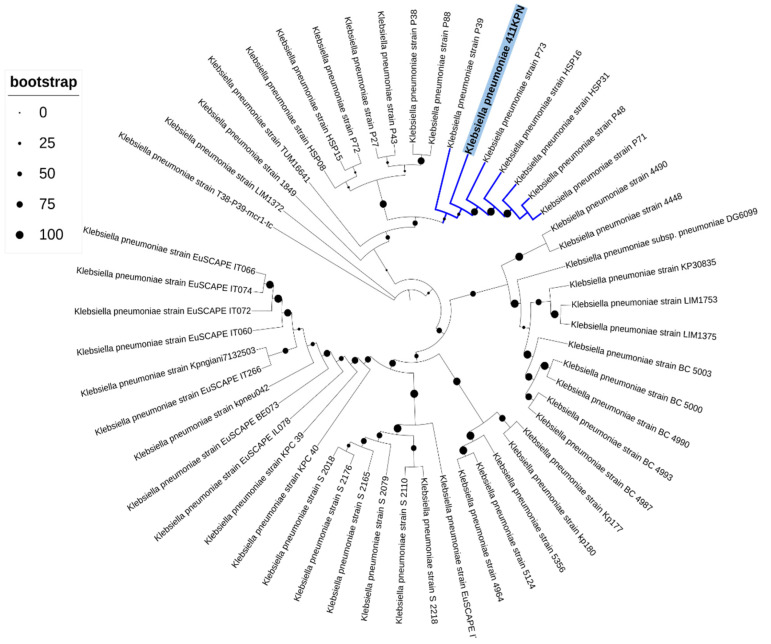
Phylogenetic tree of KP411 strain and 52 *K. pneumoniae* genomes deposited at GenBank^®^. The phylogenetic tree was built using the algorithm based on maximum likelihood (RAxML) on the PATRIC platform and edited on the iTOL v6 program. The KPN411 strain was highlighted, and the internal node is indicated in bold.

**Figure 2:**
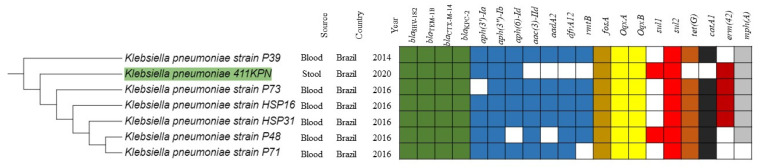
Heat map of the resistome of *Klebsiella pneumoniae* strains grouped in the phylogenetic analysis in the same cluster as the KP411 strain.

Phylogenetic analysis revealed that the KP411 strain had a greater genetic relationship with clinical isolates recovered from humans in Brazil between 2014 and 2016 (Figure 1 and Figure 2). Interestingly, all isolates of this clade had the same ARGs to β-lactams (*bla*_KPC-2_, *bla*_CTX-M-14_ e *bla*_TEM-1_) verified in the KP411 strain and a similar resistome for the other classes of antimicrobials (Figure 2).

Additionally, analyses related to the KP411 virulome demonstrated the presence of the genes *mrkABCDFHIJ* (fimbria type 3), *fimABCDEFGHIK* (fimbria type I), *iutA* (aerobactin), *entABCDEFS* (siderophere), *fepABCDG* (siderophere), and *traT* (serum resistance). These virulence factors are important for the adherence process of the bacteria to the cells at the site of infection [24]. In addition, *traT* is responsible for conferring resistance to the bactericidal activity of the human serum, which gives an important advantage to the bacterial strain, especially in cases of current bloodstream infection [25,26].

In conclusion, our findings demonstrated the occurrence of a hypermucoviscous multidrug-resistant *K. pneumoniae* ST258 strain carrying *bla*_KPC-2_ and *bla*_CTX-M-14_ genes recovered from stool samples of poultry in the Brazilian Amazon Region. Our data reveal a worrying scenario, as it shows that poultry may be acting as reservoirs of high-risk MDR clones of *K. pneumoniae* of great clinical importance, and, consequently, contributing to their dissemination.

## 3. Materials and Methods

### 3.1. Bacterial Strain

The carbapenem-resistant *K. pneumoniae* KP411 strain was recovered during a prospective surveillance study performed by the GUARANI network, which aimed to monitor the frequency of β-lactamases-producing GNB at the human–animal interface. In this study, 107 stool samples were collected from different hosts (32 humans, 30 poultry, 30 cattle, and 15 swine) from five Brazilian regions in 2020 [27]. The KP411 were isolated from the northern region and was initially retrieved in a Chromagar^TM^ Orientation plate supplemented with 2 µg of meropenem (Sigma-Aldrich, St. Louis, MO, USA).

### 3.2. In Vitro Antimicrobial Susceptibility Testing

The MICs were determined by agar dilution for the following antimicrobials (Sig-ma-Aldrich, St. Louis, MO, USA): ceftriaxone, ceftazidime, cefepime, aztreonam, ertapenem, imipenem, meropenem, gentamicin, amikacin, ciprofloxacin, and levofloxacin. The broth microdilution method was performed to determine the MICs for colistin and polymyxin B. Briefly, to perform the agar dilution, Mueller–Hinton agar (Oxoid^®^, Basingstoke, England) plates containing the antimicrobial agent to be tested were prepared in increasing concentrations. Subsequently, saline (0.85%) inoculums, adjusted to 0.5 on the MacFarland scale, were prepared from overnight culture. The bacterial inoculums were then dispensed simultaneously on the surface of the agar plates using the steers replicator (final inoculum of 5 × 10^5^ CFU/mL). The plates were incubated at 35 °C ± 1 °C for 18 ± 2 h. To perform the broth microdilution, serial dilutions of polymyxin B sulfate and colistin were prepared from a stock solution (2X) and dispensed in 96-well polystyrene microplates. Columns 11 and 12 of each microplate were used as bacterial growth and sterility control, respectively. The preparation of the solutions was carried out with cation-adjusted Mueller–Hinton broth (Oxoid, Basingstoke, England), according to the recommendations of BrCAST /EUCAST. Saline inoculums were prepared to reach the concentration of 5 × 10^5^ CFU/mL. The plates were incubated at 35 °C ± 1 °C for 18 ± 2 h. The Escherichia coli ATCC 25922 and Pseudomonas aeruginosa ATCC 2785 strains were used as quality control in both methodologies of antimicrobial susceptibility testing (AST). All results were interpreted according to the Brazilian Committee on Antimicrobial Susceptibility Testing (BrCAST/EUCAST) guidelines (http://brcast.org.br/, accessed on 15 December 2022).

### 3.3. Analysis of Hypermucoviscous Phenotype

To analyze the hypermucoviscous phenotype, 10 µL of Luria–Bertani (LB) broth (Thermo Fisher Scientific, Basingstoke, UK) with the bacterial suspension adjusted to 10^6^ CFU/mL was inoculated into blood agar plates (Laborclin, São Paulo, Brazil). The formation of a filament equal to/or greater than 10 mm was considered a positive result [28].

### 3.4. Whole-Genome Sequencing and In Silico Analysis

The DNA extraction of the KP411 strain was performed using the QIAamp DNA kit (Qiagen, Hilden, Germany) following the manufacturer’s recommendations. To prepare the DNA library, the Nextera XT kit was used (Illumina^®^, San Diego, CA, USA) and the sequencing was performed at MicrobesNG of the University of Birmingham, UK, on the Illumina^®^ HiSeq 2500 platform using 2- × 250-bp paired-end reads. To adapt and perform a quality trim of the reads, the Trimmomatic version 0.40 was used [29]. The genome was assembled using SPAdes software version 3.9.1 [30] and annotated using Prokka version 1.12 [31]. All the software used was configured with the standard mode.

The in silico detection of the ARGs was performed using ResFinder 4.1 (https://cge.cbs.dtu.dk/services/ResFinder/, accessed on 15 December 2022), the Multilocus Sequence Type (MLST) based on the Pasteur scheme was analyzed in MLST 2.0 (https://cge.cbs.dtu.dk/services/MLST/, accessed on 15 December 2022), and plasmid replicons were identified by PlasmidFinder 2.1 (https://cge.cbs.dtu.dk/services/PlasmidFinder/, accessed on 15 October 2022) in the Center for Genomic Epidemiology platform (CGE) (http://www.genomicepidemiology.org/, accessed on 15 December 2022). The association of the ARGs mediated by plasmids was performed through the binary classification of the contigs for plasmids and chromosomes using the mlplasmids tool (https://sarredondo.shinyapps.io/mlplasmids/, accessed on 15 December 2022). The typing of the polysaccharide and the lipopolysaccharide locus was performed using the Kaptive web software (https://kaptive-web.erc.monash.edu/#welcome, accessed on 15 December 2022). Virulence-encoding genes were predicted using the VFanalyzer (http://www.mgc.ac.cn/cgi-bin/VFs/v5/main.cgi?func=VFanalyzer, accessed on 15 December 2022).

To establish the genetic relationship among the KP411 and other *K. pneumoniae* strains isolated worldwide, 51 *K. pneumoniae* genomes were selected using the Similar Genome Finder service of the Pathosystems Resource Integration Center (PATRIC) [32], taking into account the following parameters: *p*-value threshold of 0.001 and distance of 0.01. The phylogenetic tree was then built employing the Codon Tree method with 1000 single-copy genes using the RAxML program on the PATRIC platform. The Interactive Tree of Life (iTOL) [33] was used for the visualization and final annotation of the phylogenetic tree.

## Figures and Tables

**Table 1:** Minimum inhibitory concentrations (MIC) of the isolate KP411 recovered from a poultry farm in the city of Castanhal, Brazil.

Antimicrobials	MIC	Interpretation
Aztreonam	128 μg/mL	R
Ceftriaxone	>256 μg/mL	R
Ceftazidime	64 μg/mL	R
Cefepime	>256 μg/mL	R
Ertapenem	256 μg/mL	R
Imipenem	64 μg/mL	R
Meropenem	64 μg/mL	R
Ciprofloxacin	>64 μg/mL	R
Levofloxacin	32 μg/mL	R
Gentamicin	64 μg/mL	R
Amikacin	4 μg/mL	S
Colistin	0.5 μg/mL	S
Polymyxin B	<0.25 μg/mL	S

**Table 2:** Nucleotide sequences comparison with IncX3 found in KP411 strain with plasmids deposited at GenBank.

Plasmid Identification	Coverage	Identity	Accession Number	Species	Source of Infection	Year	Geographical Location
pKP64477d	100%	100%	MF150120.1	*K. pneumoniae*	Urine	2014	Brazil
pKP13D	99%	100%	CP003997.1	*K. pneumoniae*	Blood	2009	Brazil
pk89	99%	99.85%	MK264770.1	*K. pneumoniae*	Rectal Swab	2015	Brazil
pk1194a	100%	99.99%	KX756453.1	*K. pneumoniae*	Catheter Tip	2011	Brazil
EC037P1	99%	99.99%	KU963389.1	*E. coli*	NI	2016	Brazil
strain KP30835 plasmid unnamed5	100%	99.96%	CP027700.1	*K. pneumoniae*	Pneumonia	2015	United States

## Data Availability

The whole-genome sequence was deposited in the GenBank database under the accession number JAOWIL000000000 BioProject PRJNA888994.
